# AutoQC-Bench: a diffusion model and benchmark for automatic quality control in high-throughput microscopy

**DOI:** 10.1038/s44303-025-00117-8

**Published:** 2025-11-07

**Authors:** Zixuan Pan, Justin Sonneck, Dennis Nagel, Anja Hasenberg, Matthias Gunzer, Yiyu Shi, Jianxu Chen

**Affiliations:** 1 Computer Science and Engineering, University of Notre Dame, Notre Dame, USA; 2 Leibniz-Institut für Analytische Wissenschaften - ISAS - e.V., Dortmund, Germany; 3 Faculty of Computer Science, Ruhr-University Bochum, Bochum, Germany; 4 Institute for Experimental Immunology, Imaging, University Hospital, University Duisburg-Essen, Essen, Germany

**Keywords:** Biological techniques, Computational biology and bioinformatics, Engineering

## Abstract

Reliable biomedical imaging demands rigorous quality control, yet high-throughput microscopy remains prone to diverse artifacts. We present AutoQC-Bench, a software based on a reconstruction-driven diffusion model flagging abnormal images without prior knowledge, and along with a benchmark of 8000 images capturing common quality issues. The software outperforms existing methods, generalizes across modalities, and supports large-scale bioimaging studies. The software and benchmark are openly shared to advance robust microscopy quality control.

## Introduction

Advanced microscopy technologies have transformed high-throughput biomedical research, enabling large-scale experiments such as drug screening and high-throughput profiling. For instance, high-throughput time-lapse imaging assays for cell migration analysis and toxicity screening can produce vast amounts of data, often comprising hundreds or thousands of movies, each with several hundreds of time frames. The fast development of artificial intelligence (AI) based microscopy image analysis has revolutionized quantitative studies at unprecedented accuracy and scale, but could still suffer from out-of-distribution (OOD)^1^ data (i.e., images with unexpected features, such as unusual lighting that significantly differs from the training data) and therefore degraded performance. Various quality issues, such as poor illumination, contamination, optical defects, out-of-focus images, etc., could compromise the dataset, despite the sophistication of modern imaging pipelines.

Ensuring data quality through effective quality control (QC) is critical for reliable downstream analysis. Traditional QC procedures typically rely on a combination of automated methods, informed by prior knowledge or heuristics (e.g., detecting intensity values outside predefined ranges), and manual selective verification. However, these semi-automated approaches can still be labor-intensive and time-consuming, and often struggle to effectively address the wide diversity of potential issues in large-scale datasets (not only imaging issues, e.g., lighting, but also sample issues, e.g, contamination), leaving considerable errors undetected. Some pilot bioimaging AI models, such as a denoising foundation model^2^ or parts of Cellpose3^3^, are able to deal with specific issues, e.g. restoring noisy microscopy images out-of-the-box to some extent, but may not be sufficient for comprehensive QC.

**Fig. 1:**
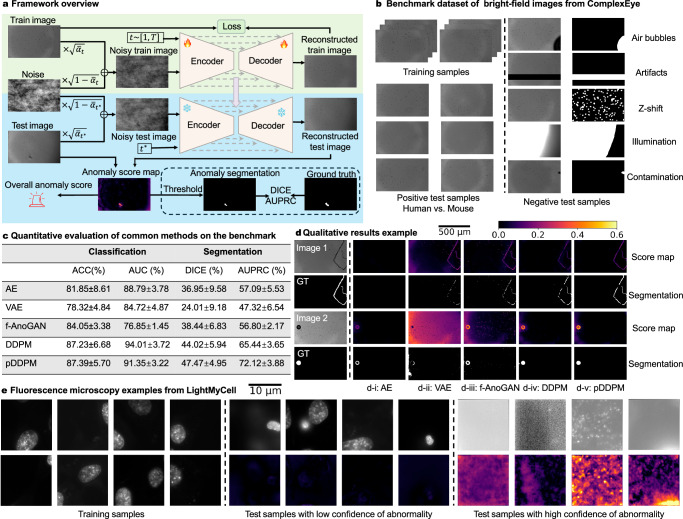
Overview of the AutoQC-Bench (software and benchmark data) and qualitative and quantitative results. **a** Schematic of the training and test workflow using a diffusion model. **b** Benchmark dataset constructed from bright-field images in ComplexEye, including training samples, positive test samples, and negative test samples with five anomaly types. **c** Quantitative performance of common methods (AE AutoEncoder, VAE Variational AutoEncoder, f-AnoGAN ^7^, DDPM Denoising Diffusion Probabilistic Model^9,10^, pDDPM ^12^) on classification and segmentation (ACC Accuracy, AUC Area Under the ROC Curve, DICE Dice similarity coefficient, AUPRC Area Under the Precision-Recall Curve). **d** Qualitative comparison of anomaly localization on two examples across methods (row 1 and 3: anomaly score map, row 2 and 4: detected anomaly area). **e** Additional experiments on fluorescence microscopy images from the LightMyCell dataset. The left section shows examples in the curated “normal” images. The middle and right sections shows examples with low and high confidence of being “bad” images, respectively (top row: raw images, bottom row: the corresponding anomaly score maps.

To address this problem, in this work, we developed a diffusion model-based automated QC toolbox (together with a new microscopy QC benchmark), which is agnostic to specific QC issues. Evaluated on both our new benchmark set and another real large-scale dataset from a public bioimaging AI challenge (LightMyCell, orginally for a different task), we show that our proposed tool can effectively flag potential quality issues and avoid false alarms on natural biological variations (see examples in Fig. 1 and more details in Methods).

The core methodology of our AutoQC tool is a reconstruction-based framework as depicted in Fig. 1a. Briefly, we train a specially designed diffusion model on a high-quality reference set with only “normal” images. When new images are collected, the model attempts to reconstruct them. If an image is “normal”, the reconstruction will closely resemble the original image. However, if an image is “abnormal”, the model will reconstruct it as if it were “normal”, since the model has only been trained on “normal” images. By comparing the discrepancy between the original and reconstructed images, we can automatically detect a wide range of potential issues, including unexpected errors. Here, the definition of “normal” images may not be the best images from an imaging perspective, but vary in different applications and may even include images containing moderate noise or artifacts. For example, a certain degree of flat-field issue due to a specific lens used in a study could have negligible effects on downstream quantitative analysis, e.g., quantifying the motility of bacteria.

While the reconstruction-based framework is not new, many different models could be used, such as the classic auto-encoder models, but the effectiveness significantly varies through our systematic evaluations. To this end, we provide a dataset of “normal” images and a comprehensive collection of flawed images from five distinct categories of issues as a benchmark for the bioimaging community. Additionally, we release the code for our method and other state-of-the-art related methods as part of a benchmarking toolkit. We hope that our work will raise the awareness of and foster further developments in automated QC, which is crucial for high-quality, large-scale downstream analyses. The overall benchmarking toolkit is presented in Fig. 1b.

We collected images for benchmarking purposes with the ComplexEye system^4^ (see details in Methods), a multi-lens array microscope designed for high-throughput immune cell migration analysis, enabling simultaneous time-lapse imaging of up to 64 wells in a 384-well plate with rapid frame acquisition. 8233 images (individual 2D frames extracted from time-lapse movies) of migrating neutrophils from human blood samples were manually curated and used as a reference set for model training. The set of images held-out for tests and benchmarking were constructed from two sources: (1) 2D image frames with imaging artifacts from a real high-throughput compound screening experiment with human blood samples, and (2) 2D image frames from short movies specially collected for this novel benchmark with mouse bone marrow samples, to represent additional normal images from different assays and additional problematic images with potential issues not in the screening experiments. In the end, the held-out evaluation set contains 100 positive samples (neutrophil images of acceptable qualities from both human blood samples and mouse bone marrow samples) and 48 negative samples with five types of issues: air bubbles, artifacts, Z-shift, illumination issues, and contamination (Fig. 1b). To enable comprehensive quantitative benchmarking, we also roughly annotated a mask for the problematic areas in all negative samples, so that the sensitivity and specificity of the model can be estimated. It is worth emphasizing that such masks are only rough delineations, different from segmentation masks, and therefore only serve as a complementary quantitative metric besides the positive/negative classification labels. In contrast to the existing dataset^5^, which provides image-level labels for supervised artefact classification in multispectral imaging patches, our dataset additionally includes pixel-level annotations of common artefacts in full-size brightfield microscopy imaging, enabling both classification and anomaly localization tasks. In practice, the accurate localization of the problematic areas could be combined with additional heuristics to further improve the QC workflow.

Quantitative results are shown in Fig. 1c with example images in Fig. 1d and additional visualizations in the Supplementary Information. It is evident that different models under the reconstruction-based framework have very different performance in identifying images with various issues. In particular, diffusion-based approaches achieved the strongest results, with pDDPM, a patch-based variant of DDPM that reconstructs each patch conditioned on its surrounding regions, performing the best. By incorporating global context while preserving local details, pDDPM improves reconstruction fidelity and enhances sensitivity to various anomalies. In our AutoQC software, this enables the model not only to detect all types of issues in the benchmark set (without prior knowledge of potential anomaly), but also generalizes well from human neutrophils to mouse neutrophils (Supplementary Fig. 2), while the problematic areas identified by the model align reasonably well with the areas highlighted by human experts.

In addition to this new benchmark set, we also tested our AutoQC software (using pDDPM) on another large-scale dataset from the public LightMyCell grand challenge (https://lightmycells.grand-challenge.org/), to evaluate the effectiveness of our method in different microscopy modalities. Briefly, the LightMyCell dataset contains over 2500 paired transmitted light images and fluorescence microscopy images of 4 different organelles in vertebrate cells to evaluate the performance of in-silico labeling models, collected from 30 different studies in the France Bioimaging Infrastructure. The scale of the data prohibited exhaustive manual or semi-automatic QC and therefore the dataset may contain various “noisy” samples in the training data. As a proof-of-concept, we take all 1289 fluorescence images of nuclei from the largest study (ID=25) and manually curated a set of 103 normal images to train our AutoQC model. Then, we applied the model to all remaining fluorescence images of nuclei. Sample images with the highest confidence and lowest confidence of being normal are presented in Fig. 1e. Our AutoQC software can provide reliable assistance in curating the large diverse dataset, which is crucial in establishing high-quality datasets for large-scale bioimaging AI competitions or for training large foundation models.

We witness a fast progress in bioimaging AI and microscopy technology in tandem. High-quality large-scale datasets play a central role, permitting innovative biomedical studies only possible at scale, and paving the path for AI researchers to establish more large foundation models for the bioimaging field. We hope that the AI-based AutoQC software we introduced in this work can help biomedical researchers to effectively reduce potential noise in their large-scale datasets, and the QC benchmark dataset we released with this work can further raise the awareness for automatic quality control in order to stimulate the development of more effective methods. We welcome contributions from the community to enlarge the benchmark set and eventually establish a community-driven standard for automatic microscopy image QC.

## Methods

### Principles of reconstruction-based quality control methods

Let ${{\bf{X}}}^{n}={\{{{\bf{x}}}_{i}^{n}\in {{\mathcal{X}}}^{n}\}}_{i = 1}^{N}$ represent the set of *N* samples on a normal data space ${{\mathcal{X}}}^{n}$, where each ${{\bf{x}}}_{i}^{n}$ is a clean image without any abnormal regions. Reconstruction-based quality control methods usually train a model *f*_*θ*_( ⋅ ) that reconstructs ${{\bf{x}}}_{i}^{n}$ from a corrupted version ${{\bf{x}}}_{i}^{n^{\prime} }$ by minimizing a reconstruction loss:

1 $$\mathop{\min }\limits_{\theta }\frac{1}{N}\mathop{\sum }\limits_{i=1}^{N}{L}_{{\rm{train}}}\left({{\bf{x}}}_{i}^{n},{\hat{{\bf{x}}}}_{i}^{n}\right),\quad \,{\text{where}}\,\,{\hat{{\bf{x}}}}_{i}^{n}={f}_{\theta }\left({{\bf{x}}}_{i}^{n^{\prime} }\right).$$

*L*_train_ is a function to measure the reconstruction quality.

When deployed, we have a test dataset with anomalies ${{\bf{X}}}^{a}={\{{{\bf{x}}}_{j}^{a}\in {{\mathcal{X}}}^{a}\}}_{j = 1}^{M}$. For any test image ${{\bf{x}}}_{j}^{a}\in {{\bf{X}}}^{a}$, we first degrade it to ${{\bf{x}}}_{j^{\prime} }^{a}$, and then use the well-trained reconstruction model ${f}_{{\theta }^{* }}(\cdot )$ to get the reconstruction ${\hat{{\bf{x}}}}_{j}^{a}$. The pixel-wise anomaly score map Λ_*j*_ is defined by the reconstruction error:

2 $${\Lambda }_{j}={L}_{{\rm{test}}}\left({{\bf{x}}}_{j}^{a},{\hat{{\bf{x}}}}_{j}^{a}\right),\quad \,{\text{where}}\,\,{\hat{{\bf{x}}}}_{j}^{a}={f}_{\theta }^{* }\left({{\bf{x}}}_{j}^{a^{\prime} }\right).$$

Here, higher values in the score map correspond to larger reconstruction errors, indicating higher probability of being abnormal. *L*_test_ serves the same purpose of assessing the reconstructed image as *L*_train_, though it may use a different function.

Once we obtain the anomaly score map Λ_*j*_, we can determine whether the test image is normal or anomalous by calculating an overall anomaly score *AS*_*j*_. In this work, we define three methods for this calculation:

**Maximum Value**: The overall anomaly score is the maximum value in Λ_*j*_:

3 $$A{S}_{j}=\max ({\Lambda }_{j}).$$

**Mean Value**: The overall anomaly score is the mean of all values in Λ_*j*_:

4 $$A{S}_{j}=\,\text{mean}\,({\Lambda }_{j}).$$

**Patch-Based Maximum**: we use a sliding window to extract overlapping patches from Λ_*j*_, compute the mean anomaly score for each patch, and select the maximum among these patch-wise mean scores as the overall anomaly score:

5 $${A}{S}_{j} = {\rm{max}}\,\left(\right.\!{\text{mean}}\left(\right.\!\Lambda_{j}^{p}\,\left)\right.\!\left)\right., \quad \forall p \in {\text{patches}}.$$

In practical applications, we can collect a small validation set containing both normal images and annotated abnormal images. To detect anomalies, we first determine an optimal threshold, denoted as AS_*_, by performing a greedy search on the validation set to best separate normal and abnormal samples. During test, the software raises an alarm if the anomaly score of a given frame exceeds this threshold, i.e., *AS*_*j*_ > AS_*_. For frames flagged as abnormal, the software then generates a pixel-wise anomaly segmentation mask to further localize the anomalous regions. To determine the optimal binarization threshold λ_*_, we conduct another greedy search on the abnormal samples by iteratively calculating Dice scores across different threshold values. The best threshold found is then used to generate the final anomaly segmentation mask *y*_*j*_, defined as:

6 $${y}_{j}(i,k)={\mathbb{I}}({\Lambda }_{j}(i,k) > {\lambda }_{* })$$

where ${\mathbb{I}}(\cdot )$ is the indicator function:

7 $${\mathbb{I}}({\Lambda }_{j}(i,k) > {\lambda }_{* })=\left\{\begin{array}{ll}1,\quad &\,\text{if}\,\,{\Lambda }_{j}(i,k) > {\lambda }_{* },\\ 0,\quad &\,\text{otherwise}\,.\end{array}\right.$$

### Baseline Methods Implemented for Benchmarking

We briefly introduce the baseline algorithms implemented in our software in this subsection.

#### AE

Autoencoders (AEs)^6^ are reconstruction-based models that consist of two main components: an encoder ${E}_{\phi }:{{\mathbb{R}}}^{H\times W\times C}\to {{\mathbb{R}}}^{d}$ and a decoder ${D}_{\theta }:{{\mathbb{R}}}^{d}\to {{\mathbb{R}}}^{H\times W\times C}$. The encoder compresses the input image ${\bf{x}}\in {{\mathbb{R}}}^{H\times W\times C}$ into a latent representation **z** = *E*_*ϕ*_(**x**), while the decoder reconstructs it from the latent code: $\hat{{\bf{x}}}={D}_{\theta }({\bf{z}})$.

The model is trained by minimizing the reconstruction error between the input **x** and its reconstruction $\hat{{\bf{x}}}$:

8 $${L}_{{\rm{AE}}}={{\mathbb{E}}}_{{\bf{x}} \sim {p}_{{\rm{data}}}}\parallel {\bf{x}}-\hat{{\bf{x}}}{\parallel }_{p},\quad \,\text{where}\,\,\hat{{\bf{x}}}={D}_{\theta }({E}_{\phi }({\bf{x}})).$$

#### VAE

Variational Autoencoders (VAEs)^6^ extend AEs by modeling the latent space probabilistically. It constrains the outputs of encoder to a Gaussian distribution in the latent space:

9 $${q}_{\phi }({\bf{z}}| {\bf{x}})={\mathcal{N}}({\mu }_{\phi }({\bf{x}}),{\sigma }_{\phi }^{2}({\bf{x}})),$$

where *μ*_*ϕ*_(**x**) and ${\sigma }_{\phi }^{2}({\bf{x}})$ are learned by the encoder. The decoder reconstructs an image from a sampled latent code:

10 $$\hat{{\bf{x}}}={D}_{\theta }({\bf{z}}),\quad {\bf{z}} \sim {q}_{\phi }({\bf{z}}| {\bf{x}}).$$

The VAE is trained to minimize the following loss:

11 $${L}_{{\rm{V\; AE}}}={{\mathbb{E}}}_{{q}_{\phi }({\bf{z}}| {\bf{x}})}\parallel {\bf{x}}-\hat{{\bf{x}}}{\parallel }_{p}+\,\text{KL}\,({q}_{\phi }({\bf{z}}| {\bf{x}})\parallel p({\bf{z}})),$$

where KL( ⋅ ) is the Kullback-Leibler divergence, and $p({\bf{z}})={\mathcal{N}}({\bf{0}},{\bf{I}})$ is a standard Gaussian prior on the latent space. VAEs ensure a smooth and structured latent space, which can help improve the generation performance.

#### f-AnoGAN

f-AnoGAN^7^ is a fast GAN-based anomaly detection method that trains a Wasserstein GAN (WGAN)^8^, consisting of a generator *G* and a discriminator *D*, exclusively on normal images. Additionally, an encoder *E* maps input images to the GAN’s latent space. The training consists of two steps: 1) Training *G* and *D* following the standard WGAN procedure with gradient penalty. 2) Training *E* by minimizing the loss ${L}_{{{\rm{izi}}}_{f}}$, which accounts for both the residual between real and reconstructed images and the residual in the discriminator’s feature space:

12 $${L}_{{{\rm{izi}}}_{f}}={{\mathbb{E}}}_{{\bf{x}} \sim {p}_{{\rm{data}}}}\left(\frac{1}{n}\parallel {\bf{x}}-G(E({\bf{x}})){\parallel }^{2}+\frac{\kappa }{{n}_{d}}\parallel f({\bf{x}})-f(G(E({\bf{x}}))){\parallel }^{2}\right),$$

where *n* is the number of image pixels, *κ* is a weighting factor, *n*_*d*_ is the dimensionality of the feature representation, and *f*( ⋅ ) represents the discriminator’s intermediate layer features.

#### DDPM

Denoising Diffusion Probabilistic Models (DDPMs)^9,10^ are a class of generative models that have recently gained significant popularity. DDPM training consists of a Markovian forward process (*diffusion process*) and a reverse sampling procedure (*reverse process*). In the *diffusion process* guided by a noise schedule ${\{{\beta }_{t}\}}_{t = 1}^{T}$, the image **x**_0_ is degraded to the noisy image **x**_*t*_ by

13 $$\begin{array}{l}q({{\bf{x}}}_{t}| {{\bf{x}}}_{0}):= {\mathcal{N}}\left({{\bf{x}}}_{t};\sqrt{{\bar{\alpha }}_{t}}{{\bf{x}}}_{0},(1-{\bar{\alpha }}_{t}){\bf{I}}\right),\\ {{\bf{x}}}_{t}=\sqrt{{\bar{\alpha }}_{t}}{{\bf{x}}}_{0}+\sqrt{1-{\bar{\alpha }}_{t}}{\boldsymbol{\epsilon }},\quad {\boldsymbol{\epsilon }} \sim {\mathcal{N}}({\bf{0}},{\bf{I}}),\end{array}$$

where *α*_*t*_ ≔ 1 − *β*_*t*_ and $${\bar{\alpha }}_{t}:= \mathop{\prod }\nolimits_{s = 1}^{t}{\alpha }_{s}$$.

^10^ show that, in the *reverse process*, we can model the distribution *p*_*θ*_(**x**_*t*−1_∣**x**_*t*_) of **x**_*t*−1_ given **x**_*t*_ as a diagonal Gaussian:

14 $${p}_{\theta }({{\bf{x}}}_{t-1}| {{\bf{x}}}_{t})={\mathcal{N}}({\mu }_{\theta }({{\bf{x}}}_{t},t),{\Sigma }_{\theta }({{\bf{x}}}_{t},t)),$$

where the mean *μ*_*θ*_(**x**_*t*_, *t*) can be calculated as a function of *ϵ*(**x**_*t*_, *t*), and the covariance *Σ*_*θ*_(**x**_*t*_, *t*) can be fixed to a known constant, following^10^. Therefore, DDPMs usually use UNet to estimate the added noise of **x**_*t*_ and optimize the network with a simple loss function:

15 $${L}_{{\rm{DDPM}}}:= {{\mathbb{E}}}_{t \sim {\mathcal{U}}(1,T),{{\bf{x}}}_{0} \sim q({{\bf{x}}}_{0}),{\boldsymbol{\epsilon }} \sim {\mathcal{N}}({\bf{0}},{\bf{I}})}\left[\parallel {\boldsymbol{\epsilon }}-{{\boldsymbol{\epsilon }}}_{\theta }({{\bf{x}}}_{t},t){\parallel }_{2}^{2}\right].$$

In the context of reconstruction-based anomaly detection, our objective is not to create new images from pure noise but to reconstruct the normal image given a noisy input image. Therefore, following^9,11,12^, we train a denoising model *f*_*θ*_(**x**_*t*_, *t*) to directly estimate **x**_0_ instead of ***ϵ***_*t*_. Another modification is that we estimate **x**_0_ from **x**_*t*_ at a fixed time step *t*_test_ when sampling, rather than *T* steps in traditional DDPM. This simplification significantly decreases the sampling time but does not affect the anomaly detection performance.

#### pDDPM

pDDPM^12^ proposes to conduct the *diffusion* and *reverse* processes only on a patch **p**_0_ while using the remaining regions as condition **c**. This technique offers better reconstruction by encouraging global context information incorporation. During evaluation, pDDPM reconstructs from noisy patches sampled over the whole image and merge all patches to one final reconstruction.

### Evaluation metrics

In this paper, we evaluate the performance of the methods using both classification (ACC and AUC) and segmentation (DICE and AUPRC) metrics.

#### ACC

Accuracy (ACC) measures the overall correctness of the classification predictions. It is defined as:

16 $$\,\text{ACC}=\frac{\text{TP}+\text{TN}}{\text{TP}+\text{TN}+\text{FP}+\text{FN}\,},$$

where TP, TN, FP, and FN represent true positives, true negatives, false positives, and false negatives, respectively. Higher ACC values indicate better overall classification performance.

#### AUC

The Area Under the ROC Curve (AUC) is used to measure the classification performance, indicating the model’s ability to distinguish between normal and anomalous samples. A higher AUC value signifies better classification performance.

#### DICE

The Dice Similarity Coefficient (DICE) evaluates the overlap between predicted segmentation and ground truth. It is defined as:

17 $$\,{\text{DICE}}\,=\frac{2| P\cap G| }{| P| +| G| },$$

where *P* and *G* are the predicted and ground truth segmentation masks, respectively. Higher DICE values indicate more accurate segmentation.

#### AUPRC

The Area Under the Precision-Recall Curve (AUPRC) measures the segmentation model’s performance in detecting anomalies at varying thresholds.

### Benchmark data acquisition with high-throughput imaging system

The ComplexEye microscope is a high-throughput multi-lens array system designed for real-time imaging of migrating immune cells. It features 16 independently controlled aberration-corrected glass lenses, each paired with a CMOS image sensor and Köhler-optimized LED illumination, enabling simultaneous imaging of 16 wells in a 96-well plate or 64 wells in a 384-well plate. The system uses a motorized XYZ stage to move the optical assembly while keeping the sample stationary, preventing motion artifacts in non-adherent cells. Imaging is performed at 4.7 × magnification with a numerical aperture (NA) of 0.3, providing a field of view of 825.8 × 512 *μ*m per well. Time-lapse imaging is captured at one frame per 8 seconds over an hour-long period, ensuring sufficient temporal resolution for automated cell tracking. The system is housed in a temperature-controlled incubator set to 37 °C to maintain optimal physiological conditions. Data acquisition and autofocus adjustments are managed by an FPGA-based controller, which optimizes image sharpness across all lenses in real time, minimizing variability between wells. This setup enables robust, high-throughput analysis of cell migration with single-cell resolution, making it ideal for large-scale screening and clinical studies.

### Sources of anomalies in benchmark dataset

#### Air bubbles

Air bubbles may occur during probe processing. Especially when pipetting small volumes such as in a 384-well plate, unwanted bubbles can occur within the well plate. When light passes those air bubbles, light will be reflected and refracted and an uneven exposure of the probe is highly likely^13^.

#### Artifacts

Artifacts can occur when dirt, most likely dust, gets into the light path, most of the parts of the microscope are not always assessable or easily cleanable. This blocks the light from passing through the probe and creates shadows and unwanted dots that might be misinterpreted as structures of interest during an automated tracking^14^.

#### Z-shift

A Z-shift can result in a blurred and unsharp image. This can occur when cells move in the Z-axis. Reasons for this can be thermodynamic changes of the media leading to movements of the cell culture media. Small inaccuracies of the microscope in finding the same Z-position reproducibly in an imaging scene can also lead to a Z-shift and a blurred image^15,16^.

#### Illumination

Illumination problems such as overexposure or uneven exposure can be caused by poor calibration or exposure time or intensity, most likely produced by a wrong Köhler illumination or a problem with the aperture^14^.

#### Contamination

When working with biological systems, even under great precautions, contaminations can occur. Biological structures such as hair, dust or mold can be introduced by the animal or the researcher into the probe itself or the microscope. This can lead to unsharp bright spots in the images^14^.

## Supplementary information

Supplementary information

## Data Availability

The benchmark dataset is released on Bioimage Archive: 10.6019/S-BIAD2133.

## Code availability

The whole project including the source code can be accessed from https://github.com/MMV-Lab/mmv_AutoQC.
