# Supplementary material for: AutoQC-Bench: a diffusion model and benchmark for automatic quality control in high-throughput microscopy
Source: Npj Imaging. 2025 Nov 7;3:57. doi: 10.1038/s44303-025-00117-8 (PMC12594752; doi:10.1038/s44303-025-00117-8)
Supplement: Supplementary file 1 — Supplementary information [file 44303_2025_117_MOESM1_ESM.pdf]

1 Implementation details

We collected 8,233 normal frames for training, with 20% of the training data used as a validation set to monitor convergence. Additionally, we gathered five types of anomalies, including air bubbles (10 frames), artifacts (8 frames), Z-shift (10 frames), illumination issues (10 frames), and contamination (10 frames), all with pixel-wise annotations for anomaly segmentation. To evaluate robustness, we perform testing over five different folds. For each fold, we randomly select 20% of the anomaly frames from only two of the five anomaly types, along with 20% of 100 additional normal samples, to form a test-time validation set for threshold selection. The remaining 80% of both normal and anomaly frames, including all five anomaly types, are used as the final test set. This setup is designed to evaluate how well thresholds tuned on limited anomaly types generalize to unseen ones. We report the mean and standard deviation of all metrics across the five folds.

All denoising-based baselines use simplex noise [1], and all models (except f-AnoGAN) employ the OpenAI U-Net or its modified variant to ensure fair comparisons. For AE, VAE and f-AnoGAN, we set the latent embedding dimension to 256. We train the models on NVIDIA A10 GPUs using the Adam optimizer (lr = 1e-4, batch size = 8) for 300 epochs. Following [2], a combination of Structural Similarity Index Measure (SSIM) and ℓ_1 loss is used to quantify the discrepancies between the reconstructions and the input.

2 Additional Ablation studies

Anomaly score calculation strategies: Supplementary Table 1 summarizes the effectiveness of different anomaly score calculation strategies, including maximum value, mean value, and patch-based maximum, on classification performance across five models (AE, VAE, f-AnoGAN, DDPM, and pDDPM). Among the three strategies, patch-based maximum yields the overall best results across all methods. Based on this observation, we adopt the patch-based maximum strategy in our final implementation.
Test timestep sensitivity: Supplementary Table 2 presents an ablation study on the effect of different test timesteps used in pDDPM. While earlier timesteps (e.g., 0) result in lower classification and segmentation performance, moderate timesteps (50 and 100) yield improved results. We also provide visual comparisons in Supplementary Figure 1. At timestep 0, abnormal regions fail to exhibit high anomaly scores compared to timestep 50, whereas excessively large timesteps introduce noise that impairs the reconstruction of normal regions. We set the test timestep as 50 to get a balance between classification and segmentation performance.
Noise type: In Supplementary Table 3, we investigate the impact of different noise types, including Gaussian noise, Coarse noise [3], and Simplex noise [1]. While standard diffusion models [4] typically rely on Gaussian noise, it underperforms in our quality control task. In contrast, both Coarse and Simplex noise lead to significantly better results, with Simplex noise achieving the best performance across most metrics. These findings highlight the critical role of structured noise in enabling more effective and reliable anomaly detection and segmentation.

Supplementary Table 1: The effectiveness of anomaly score calculation strategies on classification performance.

Method	Maximum value		Mean value		Patch-vased maximum	
	ACC (%)	AUC (%)	ACC (%)	AUC (%)	ACC (%)	AUC (%)
AE	76.97±10.57	83.96±4.78	78.32±8.00	92.27±4.58	81.85±8.60	88.79±3.78
VAE	65.55±6.93	56.03±5.74	76.30±6.81	80.91±5.32	78.32±4.84	84.72±4.87
f-AnoGAN	71.35±3.19	54.62±1.45	80.81±4.01	82.93±1.55	84.05±3.84	76.85±1.45
DDPM	84.87±3.20	90.08±3.92	82.35±10.48	95.68±4.22	87.23±6.68	94.01±3.72
pDDPM	87.06±1.84	88.78±3.26	82.86±10.37	92.81±4.33	87.39±5.70	91.35±3.22

Supplementary Table 2: Ablation studies on the test timestep.

Test timestep	ACC (%)	AUC (%)	DICE (%)	AUPRC (%)
0	83.70±6.69	88.85±4.55	41.64±5.22	62.07±4.99
50	87.39±5.70	91.35±3.22	47.47±4.95	72.12±3.88
100	87.23±5.56	90.62±2.84	48.63±4.85	72.01±3.62
200	85.55±5.86	90.96±2.89	48.88±4.92	71.42±3.41
400	85.21±5.96	91.88±3.08	48.58±5.17	70.80±3.40

Supplementary Table 3: Ablation studies on the noise type.

Noise	ACC (%)	AUC (%)	DICE (%)	AUPRC (%)
Gaussian	80.34±7.53	84.77±4.79	35.43±6.06	50.62±4.51
Coarse	86.55±8.93	91.67±4.42	39.84±4.56	58.33±4.82
Simplex	87.39±5.70	91.35±3.22	47.47±4.95	72.12±3.88

Training data purity: To evaluate the robustness of our method to training data purity, we further conducted an ablation study by contaminating the training set with anomalous images. As shown in Supplementary Table 4, while accuracy (ACC) and area under the ROC curve (AUC) remain stable across different contamination levels, the segmentation-related metrics (DICE, AUPRC) degrade more noticeably when the contamination rate increases. These results indicate that our model can tolerate a small degree of contamination in the training data, which is encouraging for real-world applications where curating a completely anomaly-free training set is difficult.

3 More experimental results

Robustness to morphological variations. To verify that our model does not incorrectly classify natural cell morphology variations as anomalies, we present additional normal test samples exhibiting diverse shapes, densities, and illumination conditions. As shown in Supplementary Figure 3, despite these visual differences, the anomaly

Supplementary Table 4: Ablation studies evaluating the impact of training data purity. "# of contamination" indicates the number of anomalous images in the training set.

# of contamination	ACC (%)	AUC (%)	DICE (%)	AUPRC (%)
0	87.39±5.70	91.35±3.22	47.47±4.95	72.12±3.88
10	87.06±3.74	91.56±3.98	47.62±4.92	61.87±4.60
20	88.57±5.69	92.15±2.86	41.21±8.78	63.36±3.32

score maps remain blank or low-activated, demonstrating that the model successfully captures the distribution of natural variations without false positives.

Cross-species generalization. Although the model was trained solely on human blood data, we also evaluated it at test data of mouse bone marrow neutrophil samples. The results in Supplementary Figure 2 show that our model generalizes well to unseen species and tissue types, producing precise and clean anomaly maps without excessive noise or missed detections. This supports the model's robustness to domain shifts in biomedical data.

Per-category anomaly examples. In Supplementary Figure 4, we further visualize representative results for each anomaly category, including air bubbles, artifacts, Z-shift, illumination issues, and contamination. These examples highlight the model's ability to detect and localize a wide range of anomalies, each with distinct spatial and appearance characteristics. The predicted maps closely match the ground-truth annotations, confirming the model's effectiveness across all error types.

Resource consumption. Supplementary Table 5 reports the training and inference time as well as GPU memory usage for all baseline models. For inference, the average time per image ranges from 3.16 s (f-AnoGAN) to 86.25 s (pDDPM), and the GPU memory consumption is relatively small (0.56–0.88 GB), which makes it feasible to integrate the algorithms into microscope control software for on-the-fly quality assessment. In practical applications, the inference speed could be further improved through techniques such as parallel processing of image patches, model quantization, or other optimization strategies, potentially allowing real-time operation that matches the microscope image acquisition speed.

References

[1] J. Wyatt, A. Leach, S. M. Schmon, and C. G. Willcocks, "Anoddpm: Anomaly detection with denoising diffusion probabilistic models using simplex noise," in *Proc. IEEE/CVF Conf. on Computer Vision and Pattern Recognition*, pp. 650–656, 2022.

[2] Z. Pan, J. Xia, Z. Yan, G. Xu, Y. Wu, Z. Jia, J. Chen, and Y. Shi, "Rethinking medical anomaly detection in brain MRI: An image quality assessment perspective," *arXiv preprint arXiv:2408.08228*, 2024.

Supplementary Table 5: Details of resource consumption, including runtime and GPU memory usage, are reported for both training and inference. Batch sizes are 8 for training and 1 for inference. We provide the average training time per epoch and the average inference time per image.

Method	Training		Inference	
	Time (s)	GPU Mem (GB)	Time (s)	GPU Mem (GB)
AE	529.23	13.15	15.79	0.56
VAE	575.65	15.42	16.95	2.82
f-AnoGAN	453.32	3.06	3.16	0.59
DDPM	759.09	17.69	22.58	0.84
pDDPM	1036.66	7.12	86.25	0.88

[3] A. Kascenas, P. Sanchez, P. Schrempf, C. Wang, W. Clackett, S. S. Mikhael, J. P. Voisey, K. Goatman, A. Weir, N. Pugeault *et al.*, "The role of noise in denoising models for anomaly detection in medical images," *Medical Image Analysis*, vol. 90, p. 102963, 2023.

[4] J. Ho, A. Jain, and P. Abbeel, "Denoising diffusion probabilistic models," in *Advances in Neural Information Processing Systems*, vol. 33, pp. 6840–6851, 2020.

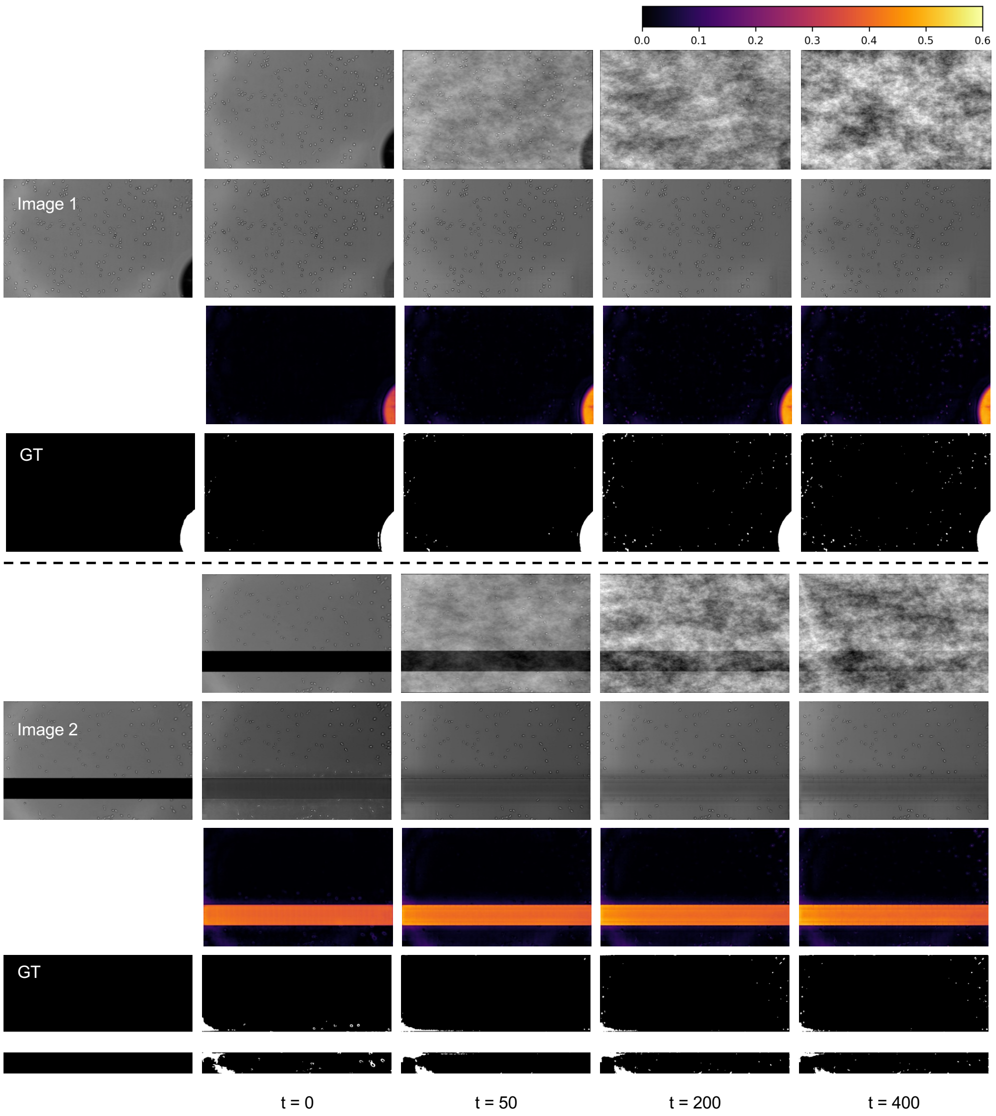

Supplementary Figure 1: Qualitative results of pDDPM using different test timesteps (row 1 and 5: the noisy images; row 2 and 6: the reconstructed images; row 3 and 7: the corresponding anomaly score maps; rows 4 and 8: the predicted anomaly masks).

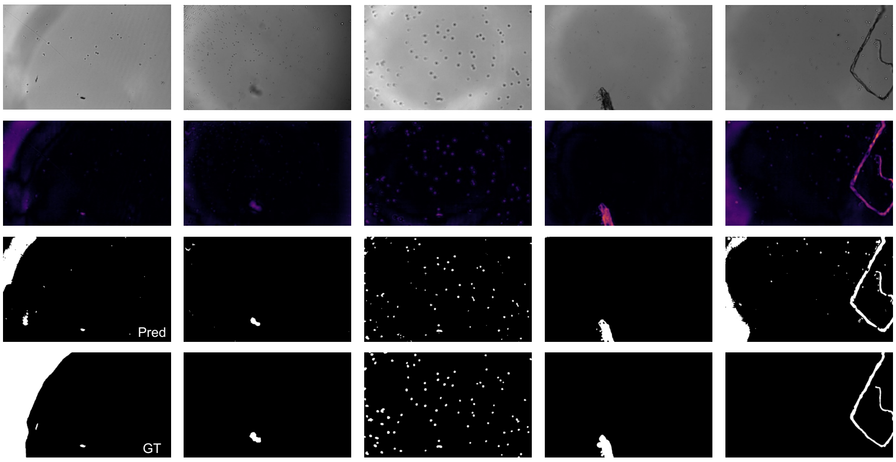

Supplementary Figure 2: Qualitative results of pDDPM on mouse neutrophil samples. Row 1 presents the raw images; row 2 shows the corresponding anomaly score maps; rows 3 and 4 illustrate the predicted anomaly masks and the ground-truth annotations, respectively.

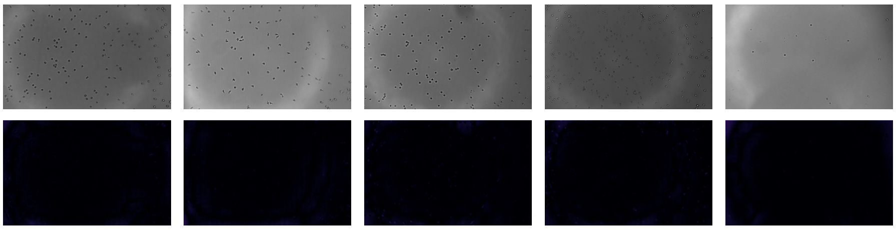

Supplementary Figure 3: Qualitative results of pDDPM on morphologically diverse normal samples. Top row presents the raw images, while bottom row shows the corresponding anomaly score maps.

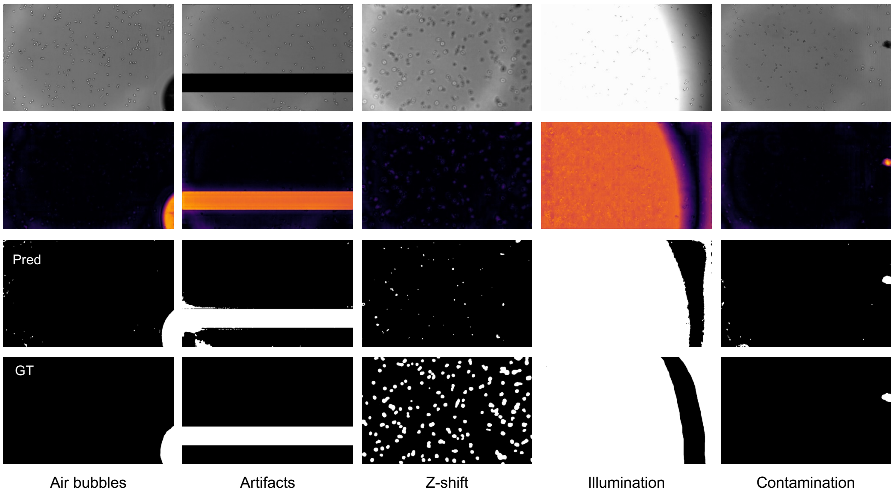

Supplementary Figure 4: Qualitative results of pDDPM across five types of anomalies. Row 1 presents the raw images; row 2 shows the corresponding anomaly score maps; rows 3 and 4 illustrate the predicted anomaly masks and the ground-truth annotations, respectively.
